# Canadian Organ Replacement Register (CORR): reflecting the past and embracing the future

**DOI:** 10.1186/s40697-014-0026-5

**Published:** 2014-10-11

**Authors:** Louise M Moist, Stanley Fenton, Joseph S Kim, John S Gill, Frank Ivis, Eric de Sa, Juliana Wu, Ahmed A Al-Jaishi, Manish M Sood, Scott Klarenbach, Brenda R Hemmelgarn, Joanne E Kappel

**Affiliations:** Department of Medicine, Division of Nephrology, University of Western Ontario, London, Ontario Canada; Lawson Health Research Institute, Kidney Clinical Research Unit, London, Ontario Canada; Department of Medicine, Division of Nephrology, University of Toronto, Toronto, Ontario Canada; Division of Nephrology, University of British Columbia, Vancouver, Canada; Canadian Institute of Health Information, Toronto, Canada; Ottawa Hospital Research Institute, The Ottawa Hospital, Ottawa, Canada; Department of Medicine, Division of Nephrology, University of Alberta, Edmonton, Alberta Canada; Department of Medicine, University of Calgary, Calgary, Alberta Canada; Department of Medicine, Division of Nephrology, University of Saskatchewan, Saskatoon, Saskatchewan Canada; London Health Sciences Centre, Victoria Hospital, Room A2-338, 800 Commissioners Road East, London, Ontario N6A 5 W9 Canada

**Keywords:** Hemodialysis, Peritoneal dialysis, Renal replacement register CORR

## Abstract

**Introduction:**

The Canadian Organ Replacement Register (CORR) is the only Canadian information system on kidney and extra-kidney organ failure and transplantation in Canada. CORR’s mandate is to record and analyze the level of activity and outcomes of vital organ transplantation and treatment of end stage kidney disease using dialysis, either hemodialysis or peritoneal dialysis, activities across Canada. The Canadian Organ Replacement Register was officially launched in 1987, and it included transplantation of extra-renal vital organs (liver, heart, lung, pancreas, bowel), in addition to renal transplantation and replacement therapy, with new financial support from the provinces.

**Objective:**

This manuscript describes the process of data acquisition and reporting, focusing on the patients with end stage kidney disease on dialysis, with data reported from the 2014 CORR Annual Data Report and the Center-Specific Reports on Clinical Measures.

**Methods:**

CORR is currently housed in the Canadian Institute for Health Information and collects data from hospital dialysis programs, regional transplant programs, organ procurement organizations and kidney dialysis services offered at independent health facilities. Data on patients is collected by completion of survey forms for each patient at the start of dialysis or receiving a transplant, using the Initial Registration form, and yearly follow up forms, which collects data on the status of the patient as of October 31^st^.

**Results:**

The incident rate per million population (RPMP) has remained stable with the exception of the 65+ age group with has experience a modest decrease since 2001. However, there has been an increasing prevalence of ESKD diagnoses, with the highest rate per million population (RPMP) amongst the age group 65+ years. This is likely attributed to gradual improving patient survival. Between 2003 and 2012, nearly 90% of dialysis patients younger than <18 and 26% of patients 75+ years survived for at least five years.

**Conclusion:**

As the number of people treated for end-stage organ failure grows, so does the importance of understanding their treatment and outcomes. In 2014, CORR continues to evolve and support the important information need to advance ESRD research and clinical practice.

**Electronic supplementary material:**

The online version of this article (doi:10.1186/s40697-014-0026-5) contains supplementary material, which is available to authorized users.

## Abrégé

**Introduction:**

Le Registre canadien des insuffisances et des transplantations d’organes (RCITO) est le seul réseau d’information sur l’insuffisance rénale et extrarénale et la transplantation au Canada. Le mandat du RCITO est de rapporter et d’analyser le niveau d’activité et les résultats des transplantations d’organes vitaux, de même que le traitement de l’insuffisance rénale terminale (IRT) par la dialyse, sous forme d’hémodialyse ou de dialyse péritonéale, au Canada. Le registre a officiellement été lancé en 1987, et il comprenait la transplantation d’organes vitaux extrarénaux (foie, cœur, poumon, pancréas, intestin), en plus de la transplantation rénale et de la thérapie de remplacement rénal, grâce à un financement nouveau des provinces.

**Objectifs:**

Le présent manuscrit décrit le processus d’acquisition et de communication des données sur les patients au stade d’insuffisance rénale terminale qui ont une thérapie de remplacement rénal, et des données tirées du rapport annuel de 2014 du RCITO et de rapports concernant les centres au sujet des mesures cliniques.

**Méthodes:**

Le RCITO est actuellement hébergé par l’Institut canadien d’information sur la santé et recueille des données au sujet des programmes de dialyse en milieu hospitalier, des programmes régionaux de transplantation, des services de prélèvement d’organes et des services de dialyse rénale offerts dans des établissements de santé indépendants. Les données sur les patients sont recueillies par le truchement d’un sondage mené auprès de chaque patient au début de la dialyse ou avant une transplantation, grâce au formulaire d’enregistrement et aux formulaires de suivi annuels, qui recueillent des données sur le statut du patient en date du 31 Octobre.

**Résultats:**

Le taux d’incidence par million de population est demeuré stable, à l’exception de la tranche d’âge des 65 ans et plus, qui a subi une faible diminution depuis 2001. Toutefois, il y a eu prévalence accrue des diagnostics d’IRT, avec le taux le plus élevé par million de population chez les 65 ans et plus. Ceci est probablement attribuable à l’amélioration graduelle de la survie des patients. Entre 2003 et 2012, près de 90% des patients en dialyse âgés de moins de 18 ans et 26% des patients de plus de 75 ans ont survécu pendant au moins 5 ans.

**Conclusion:**

L’importance de comprendre les traitements appropriés et les résultats croît à mesure qu’augmente le nombre de personnes traitées pour insuffisance d’organe. En 2014, le RCITO continue d’évoluer et de soutenir les besoins considérables en information afin de faire avancer la recherche et la pratique clinique en IRT.

## What was known before

There is a growing interest in using chronic disease registries; these data sources provide important information for policy and effective health system management, as well as for research.

## What this adds

The evolution of the Canadian Organ Replacement Register over its 40-year history has made it a valuable resource for management of kidney and extra-kidney organ failure and transplantation in Canada. Here, we describe the methodology for data acquisition and distribution and provide specific examples of the rich data that is available in the Canadian Organ Replacement Register, with a particular emphasis on end-stage kidney disease.

## Background

The Canadian Organ Replacement Register (CORR) is the only Canadian information system on kidney and extra-kidney organ failure and transplantation in Canada. CORR’s mandate is to record and analyze the level of activity and outcomes of vital organ transplantation and treatment of end stage kidney disease using dialysis, either hemodialysis (HD) or peritoneal dialysis (PD), activities across Canada. CORR provides data and information on vital organ replacement therapy, with the goal of enhancing treatment, research and patient care. Strategic advice for CORR is provided by the CORR Board of Directors, which is comprised of representatives from the Canadian Society for Nephrology, Canadian Society for Transplantation, Canadian Blood Services, Kidney Foundation of Canada, and other allied kidney health professionals. Major stakeholders include the Canadian Society of Transplantation, the Canadian Society of Nephrology, the Canadian Transplant Association, Health Canada, The Kidney Foundation of Canada, and the Canadian Association of Nephrology Nurses and Technologists.

The Canadian Institute for Health Information (CIHI) manages CORR operations, data collection, dissemination and reporting. CIHI’s mandate is to lead the development and maintenance of comprehensive and integrated health information that enables sound policy and effective health system management that improve health and health care. Although voluntary, CORR provides a comprehensive national perspective on the treatment of end organ failure in Canada. For example, CORR captures over 98% of all solid organ transplant recipients and 93% of renal dialysis patients in Canada [1].

### History of CORR

The over 40-year history of CORR can be broken into three distinct phases. In the late 1960s, Dr. Arthur Shimizu of McMaster University in Hamilton, Ontario began collecting statistics on dialysis in Ontario that would become the first Renal Failure Register. This project was so successful that it was expanded to include all of Canada by 1972, with the support of the Kidney Foundation of Canada and the Canadian Society of Nephrology. In 1973, the Register was transferred to Statistics Canada, in collaboration with the Kidney Foundation of Canada. The first report was published in 1974. In the mid-seventies, the Register was re-named to the “Canadian Renal Failure Register”, and more detailed annual reports were prepared.

In 1978, the register was decommissioned due to budgetary constraints. The register was re-started again in September 1981 under a new partnership among the Kidney Foundation of Canada, National Health and Welfare and Statistics Canada. Dr. Gerald Posen served as the project’s Medical Director, receiving guidance from the Canadian Society of Nephrology. This group’s first report was produced in 1981 and was also made available in French.

In 1987, the Register expanded, under the driving force of Dr. John Jeffrey, to include transplantation of other vital organs (liver, heart, lung, pancreas, bowel), in addition to renal transplantation and replacement therapy, with new financial support from the provinces. To reflect this change, the register was renamed the “Canadian Organ Replacement Register”. Data collection on non-renal transplant patients began formally on January 1, 1989, although some data were collected on patients transplanted prior to this date. In 1995, CORR was transferred to the Canadian Institute for Health Information (CIHI), which maintains various pan-Canadian registries and data holdings.

### CORR operation

The Canadian Institute for Health Information (CIHI) manages CORR operations, data and reporting. The current CORR team at CIHI consists of two quality assurance assistants, one data entry staff, three analysts and a program lead. The team manages all data submission and operational activities, creates analytical products, performs quality assurance, and processes data requests from researchers and other organizations. Other CIHI departments providing support to CORR include information and technology services, publications, translation, communications, privacy and legal services, and administration. CIHI receives the majority of its funding, based on a proportional model, from the provincial/territorial ministries of health and the federal government.

Subject to its privacy policy, CIHI makes its data available to enable responsive and effective health-system planning and decision-making. Researchers can request data from CORR via CIHI’s custom data request process. Data can be retrieved at an aggregate or record level. A formal Data Request form, along with a Non-Disclosure/Confidentiality Agreement (NDCA) are completed by the data requestor and submitted to CORR. The CORR team then corresponds with the data requestor and prepares detailed specifications for the request. The CORR team processes the request according to the final specifications, and makes arrangement with the requestor to ensure secure delivery of the data. For more information on CIHI’s custom data request process, please see CIHI’s Access Data page. Researchers using data from the CORR have published in top nephrology journal and altered clinical practice [2-5].

### Data sources

CORR’s target population includes all patients that received extra-renal organ transplant since January 1, 1988, and all chronic kidney failure patients who initiated renal replacement therapy (RRT) with either dialysis or transplantation as of January 1, 1981. CORR does not contain information on patients who have been determined to have acute kidney failure who require dialysis; patients with chronic kidney disease not on dialysis; recipients of tissue transplants or bone marrow transplants; patients who were listed for but did not receive a vital organ transplant; and potential organ donors (that is, deceased donors who met the criteria for donation but from whom no organs were used for transplantation). In June 2010, the CORR Board, in partnership with CIHI, initiated data collection on all newly referred ESKD patients at 16 of 18 adult kidney transplant centres across Canada. This objectives of the “Access to Kidney Transplantation Feasibility Project are to gain insights into the types of ESKD patients being referred for kidney transplant, assess the timeliness and effectiveness of wait-listing practices, and track the outcomes of patients wait-listed for deceased donor kidney transplantation. Over 7,000 patients have been recruited into this study with expected reporting of outcomes in 2015.

CORR collects data from hospital dialysis programs, regional transplant programs, organ procurement organizations (OPOs) and kidney dialysis services offered at independent health facilities. CORR receives data in a privacy-secure manner on standardized paper forms or spreadsheets. In 2011, CORR also began receiving dialysis data through electronic submissions using a recently implemented standard file format. Currently, approximately 40% of all ESKD data in CORR are submitted electronically using the new standard file format. Data submission to CORR will be fully electronic as of the 2015 data year, either using the standard file format or via a new web-based data entry tool. The Ontario Renal Network was the first organization to submit data electronically using the new standard. All data is processed at CIHI’s Toronto office. Data within the database is collected and reported on a calendar-year basis (January 1 to December 31), as is the practice in other international registries reporting on end-stage organ failure. Consistent with other national end-stage organ failure registries, CORR’s annual data report includes data from the latest calendar year for which CORR has received complete data.

This paper will describe the process of data acquisition and reporting in CORR, with a specific focus on patients with end stage kidney disease (ESKD) with data reported from the 2014 CORR Annual Data Report [6] and the Center-Specific Reports on Clinical Measures describing prevalent dialysis patients for whom CORR had received follow-up data. A second paper, in this series, will report on the patients with organ transplants [7].

### Data collection

Six forms are used to collect data on the incidence, prevalence, and outcome of all patients (adults and pediatric) with ESKD requiring dialysis, either HD or PD. These forms include: an Initial Registration, a Change of Status, a Follow-Up (Hemodialysis and Peritoneal Dialysis), and a Facility Profile form (Hemodialysis and Peritoneal Dialysis) (forms included in Additional file 1: Appendix 1 Form 1-6).

Data on ESKD patients is collected by completion of survey forms (mentioned above) for each patient at the start of dialysis, using the Initial Registration form, and a yearly follow up forms, which collects data on the status of the patient as of October 31^st^. CORR’s data is patient-oriented; when a patient is first entered into CORR, a patient identification number is assigned that will remain with the patient throughout their course of treatment. This allows for a kidney transplant record, for example, to be added to existing patient records if the patient received dialysis treatments prior to the transplant. Patients are then tracked from their first treatment for end-stage kidney disease (dialysis or transplantation) to their death, unless they become lost to follow-up or regain renal function. For the purposes of recording continuity of care, CORR captures out-of-country transfers when informed by reporting facilities.

The Initial Registration form is completed for all newly registered ESKD patients initiating dialysis. Dialysis nurses and nephrologists (and at some sites unit clerks) document patient demographic information, the primary kidney diagnosis, and existing comorbid conditions at the time of initial dialysis, in a standardized CORR form within 3 months of starting dialysis. The Change of Status form is completed for existing patients as treatment changes occur during the year. The Follow-Up form is completed for all living patients on dialysis patients on October 31^st^ of each year and includes laboratory indices, vascular access and information by modality. The Facility Profile is completed by each facility that provides dialysis to ESKD patients on December 31^st^ of each year.

CORR publishes an Annual Data Report that provides the latest data on dialysis, organ transplants, waiting list and donors. The latest report issued in 2014, reports on data from 2003–2012 and is available on CIHI’s website (http://www.cihi.ca). The Center-Specific Reports on Clinical Measures reports are new series of reports, released in 2014, designed for dialysis centres and derived primarily from the clinical measures captured in the annual follow-up survey. This includes patient demographics, comorbidities, and laboratory and clinic indicator comparisons for the center, the province and Canada.

### Data quality

Assessing the quality of CORR data is recognized as critical to ensure continued use of its data as a clinical and research tool. A validation study was conducted to assess the quality of coding of demographics, primary renal disease and comorbid conditions of patients at the start of chronic dialysis treatment compared with the patient’s medical chart (reference standard) [8]. Table 1 lists the sensitivity and specificity for each comorbidity used in CORR. We conducted a sensitivity analysis to examine if the risk of mortality would be significantly different depending on the data source used. Despite some coding issues in CORR, the direction of hazard ratios for mortality were similar whether these were calculated using the CORR data or study data obtained from chart abstraction. However, the magnitude for the hazard ratio for mortality changed for several of the risk factors.

**Table 1:** **Sensitivity and specificity of comorbid conditions within CORR compared to the medical record [** 8 **]**

**Comorbidity**	**Sensitivity (95% confidence interval)**	**Specificity (95% confidence interval)**
Angina	0.64 (0.56, 0.73)	0.91 (0.90, 0.92)
Myocardial infarction	0.62 (0.53, 0.70)	0.94 (0.91, 0.96)
Coronary artery bypass grafts/angioplasty	0.69 (0.61, 0.77)	0.97 (0.95, 0.99)
Recent history of pulmonary edema	0.62 (0.56, 0.68)	0.93 (0.91, 0.96)
Cerebrovascular disease	0.59 (0.48, 0.69)	0.96 (0.94, 0.98)
Peripheral vascular disease	0.47 (0.38, 0.55)	0.96 (0.95, 0.97)
Diabetes	0.86 (0.82, 0.90)	0.97 (0.96, 0.99)
Malignancy existing prior to first treatment	0.66 (0.57, 0.75)	0.99 (0.98, 0.99)
Chronic obstructive lung disease	0.60 (0.49, 0.70)	0.96 (0.94, 0.98)
Receiving medication for hypertension	0.89 (0.87, 0.92)	0.52 (0.39, 0.64)
Other serious illness	0.22 (0.14, 0.30)	0.93 (0.92, 0.94)
Current smoker	0.54 (0.46, 0.62)	0.95 (0.93, 0.97)

### Incident patient population

**Figure 1:**
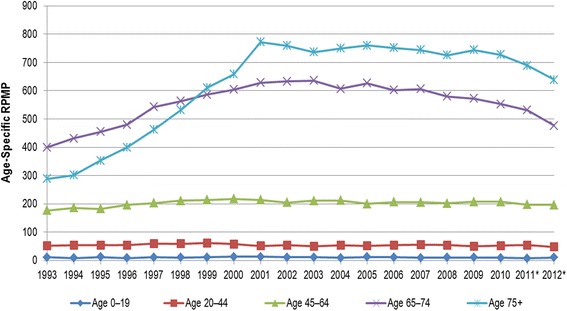
**Incident ESKD patients, age-specific rate per million population, Canada, 1993 to 2012.**

**Figure 2:**
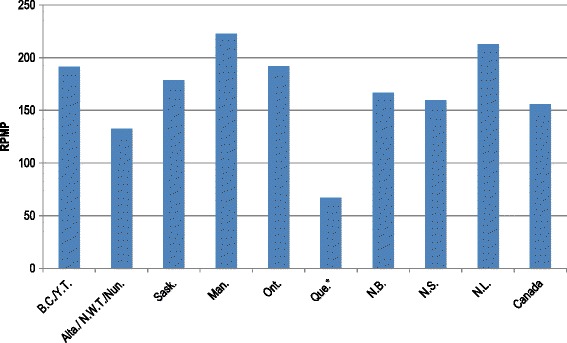
Incident ESKD patients, rate per million population by Province, Canada, 2012. *Under-reporting of incident ESKD cases from Quebec was estimated to be approximately 560 cases in 2012. This under-reporting is due to administrative issues that have temporarily halted data submission to CORR.

In 2012, there were 5,431 newly diagnosed patients with ESKD reported to CORR, almost double the number reported in 1993. The highest rate per million population (RPMP) continues to be among those age 75 and older (Figure 1), however, starting in 2005 the incidence rates among older age groups has slowly declined. Provincially, in 2012, the highest incidence occurred in Newfoundland and Labrador (212.6 RPMP) and Manitoba (222.6 RPMP) (Figure 2). Of the incident patients, 78.5% received institutional HD as their initial treatment, 11.9% Continuous Ambulatory Peritoneal Dialysis (CAPD), 5.9% Automated Peritoneal Dialysis (APD), 0.4% Home Hemodialysis (HH) and 3.4% as preemptive transplant. The proportion of patients receiving HD and PD as the initial form of treatment has been stable since 2003. The use of preemptive transplants increased over time, from 119 in 2003 to 184 in 2012. Age of incidence also influences the initial treatment. In 2012, 72% of incidence patients age 20 to 44 started with HD, while among those age 65 and older, the proportion was 84%. Pre-emptive transplant as an initial treatment was highest among younger age groups and declined with patient age. When dialysis was used to treat incident patients in 2012, all provinces used HD the majority of the time, with Newfoundland and Labrador having the highest proportion of HD (94%), followed by Saskatchewan (87%). The highest proportion of patients treated by continuous ambulatory peritoneal dialysis (CAPD) was observed in New Brunswick (20%). More than half (53%) of the newly diagnosed ESKD patients were 65 years and older and 36% were between the age of 45 and 64 years.

Select characteristics of the incident HD and PD population, from 2003–2012, are described in Table 2. In 2012, the average age of incident HD patients was 65.0, and the average age of PD patients was 62.1. Patients 65 or older accounted for 56% of incident HD patients, while males accounted for 63%.

**Table 2:** **Adult incident hemodialysis and peritoneal dialysis patients, selected characteristics, Canada, 2003 to 2012**

	**Variable**	**2003**	**2004**	**2005**	**2006**	**2007**	**2008**	**2009**	**2010**	2011*	2012*
**HD**	Mean Age (Years)	65.0	65.0	65.2	65.1	65.0	65.3	65.4	65.4	65.3	65.0
	Age 65+ (%)	58.2	57.8	59.4	57.9	57.7	57.8	58.1	57.0	57.5	56.3
	Male (%)	60.3	59.6	60.2	59.6	61.9	61.0	59.9	61.1	62.1	63.2
	Diabetes (%)^£^	44.5	45.0	46.0	47.7	49.3	48.5	49.8	51.2	53.5	53.6
	Mean Comorbidity Index^†^	2.1	2.0	2.0	2.0	2.1	2.1	2.1	2.1	2.3	2.1
	Mean BMI	27.0	27.4	27.5	27.7	27.6	28.2	28.2	28.3	28.3	28.8
	Mean eGFR^‡^	9.8	9.7	10.1	10.2	10.2	10.4	10.6	10.6	10.5	10.5
	Late Referral (%)^§^	41.7	41.3	39.5	37.7	36.0	35.7	35.9	34.8	33.6	31.4
**PD**	Mean Age (Years)	60.5	60.3	61.3	60.6	61.1	60.8	61.9	61.7	60.9	62.1
	Age 65+ (%)	45.0	42.6	47.0	44.0	45.7	44.6	45.7	46.1	43.2	46.1
	Male (%)	59.8	56.7	60.3	55.0	58.5	57.4	57.6	59.4	60.3	60.3
	Diabetes (%)^£^	41.7	43.4	45.1	43.2	43.7	42.7	44.5	47.0	45.4	51.6
	Mean Comorbidity Index^†^	1.5	1.3	1.4	1.2	1.3	1.1	1.1	1.3	1.2	1.2
	Mean BMI	26.4	26.5	26.8	27.2	27.1	27.5	28.0	27.5	27.3	27.9
	Mean eGFR^‡^	9.8	9.9	10.1	10.0	10.5	10.7	10.7	10.9	10.1	10.0
	Late Referral (%)^§^	16.2	15.8	11.4	12.2	11.3	10.4	10.1	8.6	9.1	6.7

Notes: *Under-reporting of incident ESKD cases from Quebec was estimated to be approximately 170 cases in 2011 and 560 cases in 2012.

^†^This is an adaptation of the Charlson Index for ESRD patients; it assigns each of the 14 comorbid conditions a value of 1 to 10.

^‡^Estimated glomerular filtration rate as determined by the Modification of Diet in Renal Disease (MDRD) formula (mL/min/1.73 m^2^).

^§^Patients who first see a nephrologist less than 90 days before starting dialysis.

^£^Includes cases where diabetes was reported as the primary reason for dialysis or as a co-morbidity.

HD: Hemodialysis; PD: Peritoneal dialysis; BMI: Body mass index.

A patient who starts dialysis less than 90 days after first seeing a nephrologist is considered a late referral patient. This characteristic is considered a measure of how well the early stages of kidney disease are managed. An earlier referral allows for better management of the disease and has been shown to influence patient survival [9-11]. In 2012, 27% of incident patients were late referrals, down from 37% in 2003. This improvement can be seen in all provinces. Late referral is significantly lower among patients starting PD compared to HD, and both modalities have shared a decline in late referrals (Table 2).

Using CORR data, it was identified that in Canada, as in many other countries, there has been a trend for patients to start dialysis at a higher level of kidney function or estimated glomerular filtration rate (eGFR) [2,12].A randomized controlled trial identified that starting at higher levels of eGFR (ie > 6 ml/min) without symptoms attributed to kidney failure was not associated with any improvement in clinical outcome [13,14]. Among early referral patients, that is patients who were treated by a nephrologist at least 3 months prior to initiating dialysis, the proportion of patients starting with an eGFR > 10.5 ml/min/m^2^ declined from 40.3% in 2010 to 37.1% in 2012. The mean eGFR for patients starting HD and PD has been relatively similar over time (Table 2).

BC = British Columbia; YT = Yukon; Alta = Alberta; NWT = North West Territories; Nun = Nunavut Sask = Saskatchewan; Man = Manitoba; Ont = Ontario; Que = Quebec; N.B New Brunswick; NS = Nova Scotia; NL = Newfoundland.

### Prevalent patient population

**Figure 3:**
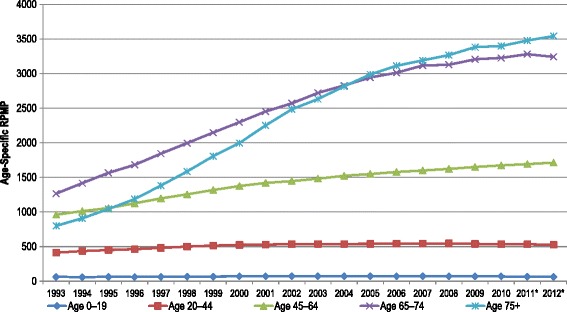
**Prevalent ESKD patients, age-specific rate per million population, Canada, 1993 to 2012.**

For CORR, prevalence is defined as the number of patients who are alive and receiving RRT (dialysis or transplant) for ESKD on December 31 of a given year. As of December 31, 2012, there were 41,252 people in Canada being treated for ESKD, with 58% (23,814) on dialysis and 42% (17,438) living with a functioning kidney transplant. Since 1993, the prevalence rate for patients being treated by dialysis has increased nearly 141%, from 283.2 RPMP to 682.7 RPMP. Figure 3 shows the rates of prevalent ESKD patients by age group. Over the 20-year period, prevalence rates increased in all age groups except among those ages 0 to 19 years. Similar to incident rates, prevalence rates in 2012 were highest in Newfoundland and Labrador and Manitoba (1,576.1and 1,559.6 RPMP). Although the incident rate among those over age 75 years has recently decreased, the prevalent rate is steadily increasing suggesting an improved survival in this age group. Hemodialysis provided in an institutional setting was the most common form of RRT across the country (45%), followed by transplant (42%). Among patients receiving dialysis 82% received HD and the remaining 18% were on PD. Among those receiving PD, 72% were using APD and the remaining on CAPD. Between 2003 and 2012, the prevalence rate of patients with diabetes as a primary diagnosis increased by 41%.

### Patient outcomes

**Figure 4:**
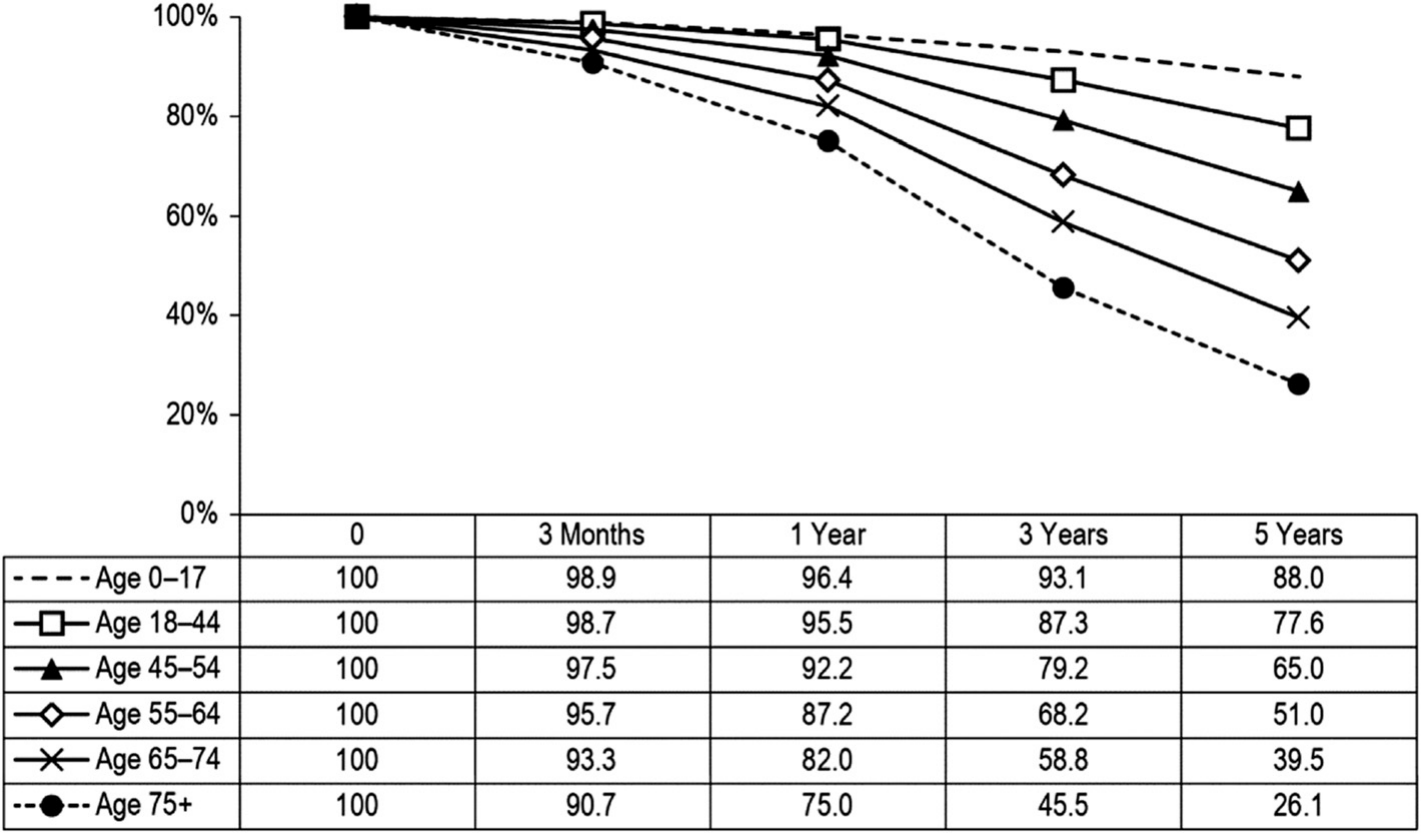
Unadjusted three month and one, three and five-year survival rates*. In Dialysis Patients, by Age Group, Canada 2003 to 2012 (percentage).

The factors associated with the survival of patients receiving dialysis treatment are well documented [5]. Long-term survival rates have been gradually improving. In general, both age and primary diagnosis affect survival of dialysis patients. Nearly 90% of dialysis patients younger than 18 survive for five years, while 26% of patients older than 75 survive for five years (Figure 4). Patients with renal vascular disease, drug-induced renal failure and diabetes have the lowest five-year survival rates, at 37%, 39% and 41%, respectively. The longest five-year survival rate is seen among patients with a primary diagnosis of polycystic kidney disease (75%) and glomerulonephritis (65%). Patients were censored at kidney transplant, lost to follow-up, left the country, or recovered kidney function. Data on survival outcomes by modality are provided in the CORR Annual Report [6].

### Center-specific report on clinical measures

In 2014, CORR developed a unique quality assessment report, the Center-Specific Report on Clinical Measures, which describes, at the facility level, data on patients included in the annual follow up survey as of October 31^st^ each year and compares facility level data to provincial and national level data. This report only includes facilities that submitted follow-up data (about 75% of prevalent patients) and excludes patients < 18 years of age. The report includes the most recent 3-year (2010 to 2012) period and compares outcomes of the individual unit to that of the province and that of Canada. The Canadian data, from this report, is described below and in Tables 3, 4 and 5.

**Table 3:** Demographics and diagnoses:* follow up hemodialysis and peritoneal patients**, Canada 2012

***Variable***		**Hemodialysis**	**Peritoneal dialysis**
***Total Patients***	*N*	14,632	3,128
***Age***	*Mean (SD)*	64.9 (15.4)	62.4 (14.9)
≥*75*	*%*	31.3	22.9
***Male***	*%*	58.7	58.5
***Race***			
*Caucasian*	*%*	63.8	61.2
*Black*	*%*	5.8	4.9
*Asian*	*%*	7.4	12.4
*Aboriginal*	*%*	6.8	6.1
*Other*	*%*	10.7	10.1
*Unknown*	*%*	5.5	5.4
***BMI***	*Mean*	28.6	27.6
*<18.5: Underweight*	*%*	3.2	2.7
*18.5–24.9: Normal*	*%*	28.2	31.6
*25.0–29.9: Overweight*	*%*	26.2	30.6
≥*30: Obese*	*%*	29.5	24.6
*Unknown*	*%*	12.9	10.4
***Primary Cause of ESKD***			
*Glomerulonephritis*	*%*	13.3	17.2
*Diabetes*	*%*	35.8	32.6
*Renal Vascular Disease*	*%*	16.5	16.8
*Polycystic Kidney Disease*	*%*	4.3	6.1
*Pyelonephritis*	*%*	4.4	3.2
*Other*	*%*	12.0	11.2
*Unknown*	*%*	13.8	12.9
***Comorbidity Index*** ^***†***^	*Mean*	1.9	1.4
***Diabetes*** ^***‡***^	*%*	47.4	42.1
***Submodality***		-	
*CAPD*	*%*	-	27.5
*APD*	*%*	-	72.5

Notes: *Data in this table is collected at the time of initial dialysis treatment for chronic renal failure.

^†^This is an adaptation of the Charlson Index for ESRD patients; it assigns each of the 14 comorbid conditions a value of 1 to 10.

^‡^Includes cases where diabetes was reported as the primary reason for dialysis or as a co-morbidity.

**Number of follow up patients differs from the number of prevalent patients reported in the CORR Annual Report (75% reported data and excludes patients < 18 years of age).

**Table 4:** Clinical ranges:* follow-up hemodialysis and peritoneal dialysis patients, per Canada 2012

***Variable***		**Hemodialysis**	**Peritoneal dialysis**
***Dialysis Adequacy***	*N*	11,380	1,759
***Urea Reduction Ratio*** ≥***65***	*%*	86.7	-
*Kt/V,* ^*†*^ *<1.2*	*%*	23.4	8.7
*Kt/V 1.2–2.0*	*%*	52.1**	14.5
*Kt/V >2.0*	*%*	2.4	33.0
*Test Not Done*	*%*	22.2	43.8
***Hemoglobin***	*N*	14,584	3,106
*<95 g/L*	*%*	11.6	11.9
*95–115 g/L*	*%*	55.3**	54.1**
*>115 g/L*	*%*	32.8	33.2
*Test Not Done*	*%*	0.3	0.7
***Transferrin Saturation***	*N*	13,827	2,829
*<20%*	*%*	25.9	20.2
*20%–50%*	*%*	64.2**	66.0**
*>50%*	*%*	4.4	4.3
*Test Not Done*	*%*	5.5	9.6
***Ferritin***	*N*	13,831	2,870
*<100 ng/mL*	*%*	13.1	18.6
*100–800 ng/mL*	*%*	64.8**	63.9**
*>800 ng/mL*	*%*	16.6	9.2
*Test Not Done*	*%*	5.5	8.2
***Target for Hgb, TSAT and FTN***	*%*	26.3	27.1
***Erythropoietin***	*%*	89.1	73.6
***Use Iron Administration***			
*No Iron*	*%*	31.4	50.8
*Oral*	*%*	12.0	43.0
*IV*	*%*	53.4	4.6
*Oral and IV*	*%*	2.9	1.0
*Unknown/Other*	*%*	0.4	0.6
***Calcium***	*N*	13,936	2,966
*<2.1 mmol/L*	*%*	11.5	6.5
*2.1–2.54 mmol/L*	*%*	69.1**	67.6**
*>2.54 mmol/L*	*%*	14.7	20.8
*Test Not Done*	*%*	4.8	5.2
***Phosphate***	*N*	14,570	3,099
*<0.80 mmol/L*	*%*	1.9	0.5
*0.80–1.45 mmol/L*	*%*	36.8**	33.9**
*>1.45 mmol/L*	*%*	60.9	64.6
*Test Not Done*	*%*	0.4	0.9
***Parathormone***	*N*	14,268	2,965
*<10.6 pmol/L*	*%*	15.4	14.4
*10.6–53.0 pmol/L*	*%*	53.6**	58.2**
*>53.0 pmol/L*	*%*	28.6	22.3
*Test Not Done*	*%*	2.5	5.2
***Target for Ca, P and PTH***	*%*	16.3	16.6
***Albumin***	*N*	14,469	3,088
*<35 g/L*	*%*	38.3	53.8
*35-40 g/L*	*%*	44.3**	36.0**
*>40 g/L*	*%*	16.2**	8.9**
*Test Not Done*	*%*	1.1	1.3
***HbA1c***	*N*	7,055	1,586
≤***7%***	*%*	61.7	62.7
***On Kidney Transplant Wait List***	*%*	21.7	34.9
***5+ Treatments/Week***	*%*	4.4	-
***15+ Hours/Week***	*%*	8.6	-

Notes: *The clinical measures reported in this table are from the annual patient follow-up as of October 31.

**Rows may be considered approximate clinical targets.

^†^Kt/V is based on URR; For HD, Kt/V is an estimation, based on the urea reduction ratio. For PD, the total weekly Kt/V (residual renal + PD) is reported.

For descriptive purposes, test values are divided into high, medium, and low.

**Table 5:** Descriptive statistics:* follow-up hemodialysis and peritoneal dialysis patients, Canada 2012

***Variable***		**Hemodialysis**	**Peritoneal dialysis**
***Kt/V*** ^**¥**^	*N*	11,380	1,759
	*Median (IQR)*	1.3 (0.3)	2.1 (0.7)
***Hemoglobin (g/L)***	*N*	14,584	3,106
	*Median (IQR))*	110.0 (6.0)	110.0 (7.0)
***Transferrin Saturation (%)***	*N*	13,827	2,829
	*Median (IQR))*	25.0 (14.0)	27.0 (15.0)
***Ferritin (ng/mL)***	*N*	13,831	2,870
	*Median (IQR)*	389.0 (510.0)	253.0 (365.0)
***Calcium*** ^***†***^ *** (mmol/L)***	*N*	13,936	2,966
	*Median (IQR)*	2.3 (0.3)	2.4 (0.2)
***Phosphate (mmol/L)***	*N*	14,570	3,099
	*Median (IQR)*	1.6 (0.6)	1.6 (0.6)
***Parathormone (pmol/L)***	*N*	14,268	2,965
	*Median (IQR)*	32.0 (43.0)	29.8 (35.0)
***Albumin*** ^***‡***^ *** (g/L)***	*N*	14,469	3,088
	*Median (IQR))*	36.0 (6.0)	34 (6.5)
***HbA1c (%)***	*N*	7,055	1,586
	*Median (IQR)*	6.6 (2.0)	6.6 (1.9)

Note: *The clinical measures in this table are from the annual patient follow-up as of October 31.

^¥^For HD, Kt/V is an estimation, based on the urea reduction ratio. For PD, the total weekly Kt/V (residual renal + PD) is reported.

^†^Calcium is corrected for albumin level. Results with ionized calcium are excluded.

^‡^Albumin reagent is unknown.

In 2012, the mean age of patients on HD was 64.9 years and patients on PD were almost 2.5 years younger (Table 3). In 2012, diabetic nephropathy accounted for the largest proportion of all prevalent patients (35%), followed by renal vascular disease (17%), and glomerulonephritis (13%). Overall, patients with diabetes (cause of ESKD or comorbidity) accounted for 47% of HD and 42% of PD patients (Table 3).

Tables 4 presents the data collected in the yearly follow up form for patients on HD and PD in 2012 and reported in the Center-Specific Report on Clinical Measures. The data are presented to highlight the proportion of patients in different ranges of indicators, with the proportion in the recommended range highlighted. The recommended ranges are taken from the Canadian Society of Nephrology guidelines or consensus statements pertaining to the care of patients on dialysis [14-17]. With the exception of serum phosphate, over 50% of HD patients fell within the recommended ranges for all clinical indicators. Compared to HD, patients being treated with PD were less likely to fall within the clinical ranges. Of note, patients receiving HD were less likely to be on the transplant waiting list compared to patients on PD (21.7 and 34.9% respectively). Table 5 presents descriptive statistics (mean or median) for select clinical variables in 2012.

### Vascular access

Table 6 presents data on the vascular access being used for HD patients between 2010 and 2012. Incident adult patients starting HD, using a catheter, increased from 77% to 81%, fistula use had a small numerical decrease from 17% to 16%, and AV graft use remained stable at approximately 1% (Table 6). Prevalent vascular access use is reported from the follow-up form. Among prevalent patients, the proportion of patients using a using the various types of HD vascular accesses remained relatively stable. Fistula use remains low compared to other jurisdictions, used in only 44.4% of patients in 2012 [18].

**Table 6:** **Vascular access use in Canada, 2010 to 2012 (percentage)**

***Variable***	**2010**	**2011**	**2012**
Incident Patients*			
*Catheter* ^*‡*^	77.2	80.9	81.1
*AV Fistula*	16.9	15.6	15.8
*AV Graft*	1.2	0.8	0.9
*Unknown*	4.7	2.6	2.1
***Prevalent Patients*** ^***†***^			
*Catheter* ^*‡*^	50.3	50.8	51.5
*AV Fistula*	45.6	45.1	44.4
*AV Graft*	3.8	4.0	3.8
*Unknown*	0.3	0.1	0.3

Notes: *Incident Patients include new adult patients who initiated hemodialysis in the year listed.

Incident access refers to access at the time of initial dialysis treatment.

^†^Prevalent Patients include adult patients for whom CORR received follow-up data by October 31^st^ in the year listed.

Prevalent access refers to the access at the time of follow-up (October 31^st^).

^‡^Includes both permanent and temporary catheters.

Catheter: Central venous catheter; AV fistula: Arteriovenous fistula; AV graft: Arteriovenous graft.

### Future directions of CORR

What started as a grass roots initiative in a single province has grown into a pan-Canadian information system that receives data from a diverse and changing group of dialysis programs, hospitals, provincial and national information systems. As part of CIHI, CORR has been able to work with this diverse group of stakeholders to maintain and provide a picture of organ replacement therapy in Canada for over 40 years. The Canadian Kidney Knowledge Translation and Generation Network (CANN-NET) has been established in partnership with the CSN (Canadian Society of Nephrology) and the KFOC (Kidney Foundation of Canada) to link Canadian kidney disease guideline producers, knowledge translation specialists and knowledge users to improve knowledge dissemination and care of patients with kidney disease. CORR data is being used to track the effectiveness of knowledge translation activity on process of care and clinical outcomes for patients with ESKD and to assist with identifying knowledge gaps in end stage kidney disease. There is a process for ongoing review of data elements, outcomes reporting and quality improvement initiatives. As the number of people treated for end-stage organ failure has grown, so has the importance of understanding their treatment and outcomes. In 2014, CORR continues to evolve to support this important need.

## Additional file

Additional file 1: Form 1. Initial Registration form. Form 2: Change of Status form. Form 3: Follow-up Hemodialysis form. Form 4: Follow-up Peritoneal dialysis form. Form 5: Facility Profile Hemodialysis form. Form 6: Facility Profile Peritoneal Dialysis form.
