# Supplementary material for: Canadian Organ Replacement Register (CORR): reflecting the past and embracing the future
Source: Can J Kidney Health Dis. 2014 Oct 11;1:26. doi: 10.1186/s40697-014-0026-5 (PMC4349772; doi:10.1186/s40697-014-0026-5)
Supplement: Additional file 1: Form 1. — Initial Registration form. Form 2: Change of Status form. Form 3: Follow-up Hemodialysis form. Form 4: Follow-up Peritoneal dialysis form. Form 5: Facility Profile Hemodialysis form. Form 6: Facility Profile Peritoneal Dialysis form. [file 40697_2014_26_MOESM1_ESM.pdf]

SEND THIS CONFIDENTIAL INFORMATION TO:
Canadian Organ Replacement Register (CORR)
Canadian Institute for Health Information
4110 Yonge Street, Suite 300
Toronto, ON M2P 2B7
Tel: 416-481-2002

Form 1: Initial Registration form

Hospital Name: ______________________________________

Hospital City: ______________________________________

Hospital Number: ______________________________________

SECTION A—PERSONAL IDENTIFICATION

(Patient label may be attached if same information is provided.)

Patient Last Name: ______________________________________

Patient Former Name: ______________________________________

Patient First and Middle Names: ______________________________

Patient Address *(city and province only)*: ______________________

Patient Postal Code: |___|___|___| |___|___|___|

Health Card Number: ______________________________________

Province of Health Card: ______________________________________

Date of Birth: |___|___|/|___|___|___|/|___|___|___|___| (DD/MON/YYYY)

Sex *(check one)*: ☐ Male ☐ Female ☐ Other

Race *(check one)*: ☐ Caucasian/white (01) ☐ Asian (02)

☐ Black (03) ☐ Indian Sub-continent (05) ☐ Pacific Islander (08)

☐ Aboriginal (09) ☐ Mid-East/Arabian (10) ☐ Latin American (11)

☐ Unknown (98)________________ ☐ Other/Multiracial (99) ______________

Affix patient label, if available.

SECTION B—PRE-DIALYSIS AND INITIAL BLOOD WORK

Date when patient first seen by nephrologist:

|___|___|/|___|___|___|/|___|___|___|___| (DD/MON/YYYY)

Was patient followed by a nephrologist prior to initiating dialysis?

(check one):
- ☐ no pre-dialysis follow-up (0)
- ☐ yes followed in nephrologist's office (1)
- ☐ yes followed in speciality clinic (2)
- ☐ yes followed in both office and clinic (3)
- ☐ unknown (9)

Was the patient receiving erythropoietin (i.e. Eprex, Aranesp) prior to initial dialysis treatment?

☐ no ☐ yes ☐ unknown

If yes:

☐ Eprex ☐ Aranesp ☐ Other

Last blood work before initial dialysis treatment: *(Indicate NA if not available)*

Haemoglobin (g/L) __________ Creatinine (μmol/L) ______________

Urea (mmol/L) _____________ Serum Bicarbonate/CO_2 (mmol/L) ________

Serum Calcium (mmol/L) ________ ☐ uncorrected ☐ corrected ☐ ionized

Serum Phosphate (mmol/L) __________ Serum Albumin (g/L) ____________

Serum Parathormone (PTH) __________ ☐ pmol/L ☐ ng/L ☐ pg/ml

SECTION C—INITIAL AND INTENDED DIALYSIS TREATMENT

Access used at time of initial dialysis *(check one)*:

Haemodialysis ☐ Temporary catheter non-cuffed (1)

☐ Temporary catheter cuffed (2) ☐ Permanent catheter non-cuffed (3)

☐ Permanent catheter cuffed (4) ☐ AV fistula (5) ☐ AV graft (6)

Peritoneal Dialysis ☐ PD catheter (7)

Date of first renal replacement therapy:

|___|___|/|___|___|___|/|___|___|___|___| (DD/MON/YYYY)

Initial dialysis treatment type *(Specify location, type and level of assistance/care.)*

LOCATION:
☐ Acute care hospital ☐ Chronic care hospital ☐ Community centre ☐ Home

TYPE:
☐ Conventional haemo ☐ Short daily haemo ☐ Slow nocturnal haemo

☐ CAPD ☐ APD ☐ Peritoneal dialysis combined with haemo

ASSISTANCE/CARE:
☐ Total care ☐ Limited self care ☐ Total self care

Is this initial treatment the intended long-term dialysis treatment for this patient?

☐ Unknown

☐ Yes

☐ No 〈 If not, why not?
- ☐ no facilities/space available (1)
- ☐ no mature access (2)
- ☐ unforeseen change in patient status leading to sudden dialysis start (3)
- ☐ other (specify) ______________________ (4)

If not, what is the long-term intended treatment for this patient? *(Specify location, type and level of assistance/care.)*

LOCATION:
☐ Acute care hospital ☐ Chronic care hospital ☐ Community centre ☐ Home

TYPE:
☐ Conventional haemo ☐ Short daily haemo ☐ Slow nocturnal haemo

☐ CAPD ☐ APD ☐ Peritoneal dialysis combined with haemo

ASSISTANCE/CARE
☐ Total care ☐ Limited self care ☐ Total self care

Patient Last Name: ______________________________

SECTION D—HEIGHT AND WEIGHT

Height/weight cannot be provided because patient is:

☐ A double-leg amputee

Record patient's height (cm) at the start of the first dialysis treatment this year:

|___|___|___|•|___|___|___| cm

Record patient's actual weight (kg) within the first month of treatment:

|___|___|___|•|___|___|___| kg

Conversion factors: 1 inch = 2.54 cm; 1 lb = 0.454 kg

SECTION E—PRIMARY DIAGNOSIS AND RISK FACTOR HISTORY

Primary renal disease *(see codes on page 3)*: |___|___|

Specify: ______________________________

Risk Factors/Co-morbid Conditions ***(check one response per condition)*****:**

	No	Yes	Unknown
a) Angina	☐	☐	☐
b) Myocardial infarct	☐	☐	☐
c) Coronary artery bypass grafts/angioplasty	☐	☐	☐
d) Recent history of pulmonary edema *(i.e. episode(s) of congestive heart failure or pulmonary edema within 6 months prior to dialysis)*	☐	☐	☐
e) Cerebrovascular disease *(i.e. stroke, transient ischemic attack, carotid surgery)*	☐	☐	☐
f) Peripheral vascular disease *(i.e. previous surgery such as femoropopliteal bypass graft, iliac or femoral endarterectomy, angioplasty, etc.; ischemic muscle pain precipitated by exercise; ischemic ulcers; gangrene; amputation)*	☐	☐	☐
g) Diabetes Type 1	☐	☐	☐
h) Diabetes Type 2	☐	☐	☐
i) Malignancy existing prior to first treatment	☐	☐	☐

If yes, indicate site using the codes listed on page 3 or specify:

|___|___| ______________________________

	No	Yes	Unknown
j) Chronic obstructive lung disease *(i.e. emphysema or chronic bronchitis)*	☐	☐	☐
k) Receiving medication for hypertension	☐	☐	☐
l) Other serious illness that could shorten life expectancy to less than 5 years	☐	☐	☐

If yes, specify condition: ______________________________

____

	No	Yes	Unknown
m) Current smoker *(i.e. has smoked cigarettes, cigars or a pipe in the last three months)*	☐	☐	☐

Treatment Codes

Consists of treatment location, treatment type and level of assistance/care required.

LOCATION

1 = Acute Care Hospital: Treatments carried out in a dialysis facility located in or on the grounds of a hospital that provides full renal care services (i.e. services provided under the care of nephrologist(s), which include social work and dietary consultation and inpatient back-up care).

2 = Chronic Care Hospital: Treatments carried out in a facility where ongoing medical intervention is provided and residents require assistance. Includes chronic care facilities and nursing homes.

3 = Community Centre: Dialysis done outside a hospital. Treatment may occur in an office building, shopping plaza or other place where nephrology inpatient services are not onsite. This includes mobile dialysis services, and dialysis provided at independent health facilities.

4 = Home: Treatments carried out in the patient's home by the patient and/or family member(s).

TYPE

1 = Conventional Haemodialysis: Given 3–6 hours two to four times a week.

2 = Short Daily Haemodialysis: Given during the day or evening for 2–3 hours 5 to 7 days per week.

3 = Slow Nocturnal Haemodialysis: Given 5–6 nights per week.

4 = CAPD (Continuous Ambulatory Peritoneal Dialysis): Patient receives peritoneal dialysis treatments through an implanted peritoneal catheter continuously throughout the day and night.
The fluid held in the abdominal cavity is exchanged an average of 4 times per 24 hours, with a usual volume of 2 litres (includes enhanced CAPD).

5 = APD (Automated Peritoneal Dialysis): An automated cycler is used to effect the dialysate exchanges while the patient sleeps at night with or without additional exchanges during the day. Excludes night manual exchanges and non-automated night exchanges.

6 = Peritoneal Dialysis Combined with Haemodialysis: Patient is receiving a combination of any type of peritoneal dialysis and haemodialysis.

For Type of Treatment code 6 only, location and level of assistance are to be coded as 0 (i.e. 0-6-0).

ASSISTANCE/CARE REQUIRED

1 = Total Care: Patient is under the full care of trained staff affiliated with a nephrology unit.

2 = Limited Self Care: Patient receives a minimal amount of assistance from trained staff affiliated with a nephrology unit.
This does not include family member(s).

3 = Total Self Care: Patient is completely responsible for his/her own treatment, with no assistance from nephrology trained staff.
A patient may be classified as total self care if he/she receives assistance from family member(s) or home care worker who is not a trained staff affiliated with a nephrology unit.

Examples:

An elderly, infirmed patient waiting for a chronic care bed but being treated at an acute care hospital with conventional haemodialyis would be coded: |1|1|1|.

A patient on short daily haemodialysis who is being treated at the acute care hospital with only some care provided by trained staff would be coded: |1|2|2|.

A patient on home CAPD receiving no assistance from trained staff would be coded: |4|4|3|.

Formulaire disponible en français.

Site of Primary Malignancy Codes

11 Two or more primary malignancies

SKIN (excludes lips and genitals)

20 Squamous cell carcinoma
21 Basal cell carcinoma
22 Squamous and basal cell carcinoma
23 Malignant melanoma

LEUKAEMIAS AND RETICULOSES

25 Myeloma
26 Acute leukaemia
27 Chronic leukaemia
29 Reticulum cell sarcoma
30 Kaposi sarcoma
31 Lymphosarcoma
33 Plasma cell lymphoma
34 Hodgkin's disease
35 Lymphoreticular tumours
36 Histiocytic reticulosis

GASTRO-INTESTINAL TRACT

40 Lip
41 Tongue
42 Parotid
43 Oesophagus
44 Stomach
45 Colon
46 Rectum
47 Anus
48 Liver—primary hepatoma
49 Liver—primary lymphoma
50 Gallbladder and bile duct
51 Pancreas

NECK AND THROAT

53 Larynx
54 Thyroid
55 Bronchus
56 Lung, primary tumour

UROGENITAL TRACT

60 Kidney—Wilms' tumour
61 Kidney—Hypernephroma of host kidney
62 Kidney—Hypernephroma of graft kidney
63 Renal pelvis
64 Ureter
65 Urinary bladder
66 Urethra
67 Prostate
68 Testis
69 Penis
70 Scrotum
71 Perineum
72 Vulva
73 Vagina
74 Uterus—cervix
75 Uterus—body
76 Ovary

MISCELLANEOUS

80 Breast
81 Muscle
82 Bone
83 Brain—primary lymphoma
84 Brain—other primary tumour
85 Other tumour of central nervous system
90 Metastatic carcinoma, primary site unknown
99 Other primary tumour, specify ____________

____

Primary Renal Diagnosis Codes

00 Chronic renal failure—aetiology uncertain

GLOMERULONEPHRITIS/ AUTOIMMUNE DISEASES

05 Mesangial proliferative glomerulonephritis
06 Minimal lesion glomerulonephritis
07 Post-strep glomerulonephritis
08 Rapidly progressive glomerulonephritis
09 Focal glomerulonephritis (adults)
10 Glomerulonephritis, histologically NOT examined
11 Severe nephrotic syndrome with focal sclerosis (paediatric patients)
12 IgA nephropathy—proven by immunofluorescence (not code 85)
13 Dense deposit disease—proven by immunofluorescence and/or electron microscopy (MPGN Type II)
14 Membranous nephropathy
15 Membranoproliferative mesangiocapillary glomerulonephritis (MPGN Type I)
16 Idiopathic crescentic glomerulonephritis (diffuse proliferative)
17 Congenital nephrosis or congenital nephrotic syndrome
19 Glomerulonephritis, histologically examined—specify

____

73 Polyarteritis
74 Wegener's granulomatosis
84 Lupus erythematosus
85 Henoch-Schonlein purpura
86 Goodpasture's syndrome
87 Scleroderma
88 Haemolytic uraemic syndrome

NEPHROPATHY, DRUG-INDUCED

30 Nephropathy caused by drugs or nephrotoxic agents—cause not specified
31 Nephropathy due to analgesic drugs
32 Nephropathy due to cisplatin
33 Nephropathy due to Cyclosporin A
39 Nephropathy caused by other specific drugs—specify ______________________

POLYCYSTIC KIDNEY

41 Polycystic kidneys, adult type (dominant)
42 Polycystic kidneys, infantile and juvenile types (recessive)

DIABETES

80 Diabetic nephropathy associated with Type 1
81 Diabetic nephropathy associated with Type 2

CONGENITAL/HEREDITARY RENAL DISEASES

21 Pyelonephritis/Interstitial nephritis associated with neurogenic bladder
22 Pyelonephritis/Interstitial nephritis due to congenital obstructive uropathy with or without vesico-ureteric reflux
24 Pyelonephritis/Interstitial nephritis due to vesico-ureteric reflux without obstruction
40 Cystic kidney disease, type unspecified
41 Polycystic kidneys, adult type (dominant)
42 Polycystic kidneys, infantile and juvenile types (recessive)
43 Medullary cystic disease, including nephronophthisis
49 Cystic kidney disease, other type—specify

____

50 Hereditary/familial nephropathy, type unspecified
51 Hereditary nephritis with nerve deafness (Alport's Syndrome)
52 Cystinosis
53 Oxalosis
54 Fabry's disease
55 DRASH syndrome
58 Posterior urethral valves
59 Hereditary nephropathy, other—specify

____

60 Congenital renal hypoplasia, specify

____

61 Oligomegonephronic hypoplasia
62 Segmental renal hypoplasia (Ask-Upmark kidney)
63 Congenital renal dysplasia with or without urinary tract malformation
66 Syndrome of agenesis of abdominal muscles (Prune belly syndrome)

RENAL VASCULAR DISEASE

70 Renal vascular disease, type unspecified
71 Malignant hypertension (no primary renal disease)
72 Renal vascular disease due to hypertension (no primary renal disease)
73 Polyarteritis nodosa
78 Atheroembolic renal disease
79 Renal vascular disease, classified (nephrosclerosis, renal vascular thrombosis)

OTHER

20 Pyelonephritis/Interstitial nephritis, cause not specified
23 Pyelonephritis/Interstitial nephritis, due to acquired obstructive uropathy—specify

____

25 Pyelonephritis/Interstitial nephritis, due to urolithiasis
29 Pyelonephritis, other causes
56 Sickle cell nephropathy
57 Wilms' tumour
82 Multiple myeloma
83 Amyloid
89 Multi-system disease, other—specify

____

90 Cortical or acute tubular necrosis
91 Tuberculosis
92 Gout
93 Nephrocalcinosis and hypercalcaemic nephropathy
94 Balkan nephropathy
95 Kidney tumour
96 Traumatic or surgical loss of kidney
97 HIV nephropathy
99 Other identified renal disorder—specify

____

SEND THIS CONFIDENTIAL INFORMATION TO:
Canadian Organ Replacement Register (CORR)
Canadian Institute for Health Information
4110 Yonge Street, Suite 300
Toronto, ON M2P 2B7
Tel.: 416-481-2002

Form 2: Change of Status form

Hospital name: ______________________________

Hospital city: ______________________________

Hospital number: ______________________________

SECTION A—PERSONAL IDENTIFICATION

(Patient label may be attached if same information is provided.)

Patient last name: ______________________________

Patient first and middle names: ______________________________

Health card number: ______________________________

Prov. or terr. of health card: ______________________________

Date of birth: |__|__|/|__|__|__|/|__|__|__|__| (DD/MON/YYYY)

Affix patient label, if available.

SECTION B—TREATMENT AND CHANGES

Record treatment changes for this patient during the calendar year, including transfers. When applicable, circle appropriate transfer, withdrew *and* died *codes in column one. Please enter name and city of hospital for each transfer-in and transfer-out. All treatment/transfer change codes are listed below and are defined on the reverse.* ***Treatment location, type and level of assistance/care must be specified when there is a treatment change.***

	(Circle code.)	Treatment Location Type Care (See codes below.)	DD	MON	YYYY	Hospital Name	Hospital City	Major Reason for Change (See codes below.)	Specify, if other.
1st treatment		|___|___|___|	|__|__|	|__|__|__|	|__|__|__|__|	________	________		
1st change	T R W D	|___|___|___|	|__|__|	|__|__|__|	|__|__|__|__|	________	________	|__|__|	________
2nd change	T R W D	|___|___|___|	|__|__|	|__|__|__|	|__|__|__|__|	________	________	|__|__|	________
3rd change	T R W D	|___|___|___|	|__|__|	|__|__|__|	|__|__|__|__|	________	________	|__|__|	________
4th change	T R W D	|___|___|___|	|__|__|	|__|__|__|	|__|__|__|__|	________	________	|__|__|	________
5th change	T R W D	|___|___|___|	|__|__|	|__|__|__|	|__|__|__|__|	________	________	|__|__|	________

If transplant, specify organ(s): ______________________________

Transfer/Withdrew/Died Change Codes *(See reverse for definitions).*

T	Transfer in	R	Transfer out
W	Withdrew from treatment *(Complete Sections D or C, where applicable.)*	D	Died *(Complete Sections C or D, where applicable.)*

Treatment Codes *(See reverse for definition).*
Consists of treatment location, treatment type and level of assistance/care required.

LOCATION	TYPE	ASSISTANCE/CARE
1 Acute care hospital	1 Conventional hemodialysis	1 Total care
2 Chronic care hospital	2 Short daily hemodialysis	2 Limited self care
3 Community centre	3 Slow nocturnal hemodialysis	3 Total self care
4 Home	4 Continuous ambulatory peritoneal dialysis (CAPD)	
	5 Automated peritoneal dialysis (APD)	
	6 Peritoneal dialysis combined with hemodialysis (code 060)	
	7 Transplantation (code 171)	

Examples:
An elderly, infirm patient waiting for a chronic care bed but being treated at an acute care hospital with conventional hemodialysis would be coded |1|1|1|.

A patient on short daily hemodialysis, who is being treated at the acute care hospital with only some care provided by trained staff, would be coded |1|2|2|.

A patient on home CAPD receiving no assistance from trained staff would be coded |4|4|3|.

Major Reason for Treatment Change Codes

HD Specific		Other	
15	Hemodialysis access failure	03	Inadequate dialysis
17	Cardiovascular instability	08	Transferred to originally intended treatment
**PD Specific**		14	Patient/family unable to cope with current treatment (patient/family initiated change)
01	Peritonitis	18	Resource/geographical (non-medical)
02	Other abdominal complications	09	Transplanted
16	Other complications related to PD	10	Recovered function
		19	Failed transplant
		11	Lost to follow-up
		20	Left country
		99	Other, specify ________

SECTION C—CAUSE OF DEATH

If patient died, enter the cause of death. (See reverse for codes.) |___|___|

Specify, if other: ______________________________

Date of death: |__|__|/|__|__|__|/|__|__|__|__| (DD/MON/YYYY)

SECTION D—REASON FOR WITHDRAWAL

If this patient has withdrawn from renal replacement therapy (even if he/she has died), please check the major reason for withdrawal:

- ☐ Psychosocial (1)
- ☐ Vascular (stroke, peripheral vascular disease, etc.) (2)
- ☐ Heart disease (3)
- ☐ Infection (4)
- ☐ Cancer (5)
- ☐ Dementia (6)
- ☐ Other (specify) ______________________________(7)
- ☐ Palliative care (8)
- ☐ Unknown (9)

Canadian Institute for Health Information
Institut canadien d'information sur la santé

Formulaire disponible en français.

Form CSD2014

Treatment Codes

Consists of treatment location, treatment type and level of assistance/care required.

LOCATION

1 = Acute care hospital: Treatments carried out in a dialysis facility located in or on the grounds of a hospital that provides full renal care services (i.e. services provided under the care of nephrologists, including social work, dietary consultation and inpatient back-up care).

2 = Chronic care hospital: Treatments carried out in a facility where ongoing medical intervention is provided and residents require assistance. Includes chronic care facilities and nursing homes.

3 = Community centre: Dialysis done outside a hospital. Treatment may occur in an office building, shopping plaza or other place where nephrology inpatient services are not onsite. This includes mobile dialysis services and dialysis provided at independent health facilities.

4 = Home: Treatments carried out in the patient's home by the patient and/or family member(s).

TYPE

1 = Conventional hemodialysis: Given 3 to 6 hours 2 to 4 times a week.

2 = Short daily hemodialysis: Given during the day or evening for 2 to 3 hours 5 to 7 days per week.

3 = Slow nocturnal hemodialysis: Given 5 to 6 nights per week.

4 = CAPD (continuous ambulatory peritoneal dialysis): Patient receives peritoneal dialysis treatments through an implanted peritoneal catheter continuously throughout the day and night. The fluid held in the abdominal cavity is exchanged an average of 4 times per 24 hours, with a usual volume of 2 litres (includes enhanced CAPD).

5 = APD (automated peritoneal dialysis): An automated cycler is used to affect the dialysate exchanges while the patient sleeps at night with or without additional exchanges during the day. Excludes night manual exchanges and non-automated night exchanges.

6 = Peritoneal dialysis combined with hemodialysis: Patient is receiving a combination of any type of peritoneal dialysis and hemodialysis (code 060).

7 = Transplantation (code 171).

ASSISTANCE/CARE REQUIRED

1 = Total care: Patient is under the full care of trained staff affiliated with a nephrology unit.

2 = Limited self care: Patient receives a minimal amount of assistance from trained staff affiliated with a nephrology unit.

3 = Total self care: Patient is completely responsible for his/her own treatment, with no assistance from trained nephrology staff. A patient may be classified as total self care if he/she receives assistance from family members or a home care worker who is not a trained staff member affiliated with a nephrology unit.

Cause of Death Codes

GENERIC

00 Cause of death uncertain, not determined

ACCIDENT

81 Accident related to treatment
82 Accident unrelated to treatment

CARDIAC

11 Myocardial ischemia and infarction
12 Hyperkalemia
13 Hemorrhagic pericarditis
14 Other causes of cardiac failure
15 Cardiac arrest, cause unknown
16 Hypertensive cardiac failure
17 Hypokalemia
18 Fluid overload

GASTROINTESTINAL

02 Gastrointestinal tumour with or without perforation
20 Acute gastroenteritis with dehydration
23 Gastrointestinal hemorrhage
29 Mesenteric infarction
62 Pancreatitis
68 Perforation of peptic ulcer
70 Sclerosing (or adhesive) peritoneal disease
72 Perforation of colon/small bowel

HEMATOLOGIC

63 Bone marrow depression
71 Thrombocytopenia
73 Thrombosis—specify ______________________

INFECTION

03 Infection (bacterial)—specify site _____________
04 Infection (viral)—specify site ________________
05 Infection (fungal)—specify site _______________
06 Cytomegalovirus
07 Epstein-Barr virus
08 Pneumocystis carinii pneumonia (PCP)
09 Protozoal/parasitic infection (includes toxoplasmosis)
10 Wound infection—specify site ______________
34 Infections elsewhere (except viral hepatitis codes 41 and 42)
35 Septicema/sepsis—specify source ______________________________________
36 Tuberculosis (lung)
37 Tuberculosis (elsewhere)
38 Generalized viral infection—specify viral agent ______________________________
39 Peritonitis (not code 70)

LIVER

41 Liver, due to hepatitis B virus
42 Liver, due to other viral hepatitis
43 Liver, drug toxicity—specify drug ________________________
44 Cirrhosis, not viral
45 Cystic liver disease
46 Liver failure, cause unknown
74 Liver, due to hepatitis C virus

METABOLIC

59 Drug-related toxicity—specify drug ____________________________

NEUROLOGIC

75 Drug neurotoxicity—specify drug ___________
76 Status epilepticus
77 Neurologic infection—specify infectious agent ________________________

RENAL DISEASE

61 Uremia caused by kidney transplant failure

RESPIRATORY

19 Acute respiratory distress syndrome (ARDS)
31 Pulmonary infection (bacterial)
32 Pulmonary infection (viral)
33 Pulmonary infection (fungal)
49 Bronchiolitis obliterans

SOCIAL

50 Drug abuse (excludes alcohol abuse)
51 Patient refused further treatment
52 Suicide
53 Therapy ceased for any other reason
54 Alcohol abuse

VASCULAR

21 Pulmonary embolus
22 Cerebrovascular accident
24 Hemorrhage from graft site—specify ________
25 Hemorrhage from vascular access or dialysis circuit
26 Ruptured vascular aneurysm (not codes 22 or 25)
27 Hemorrhage from surgery (not codes 23, 24 or 26)—specify ______________________
28 Other hemorrhage (not codes 23 to 27)
55 Vascular thrombosis
56 Pulmonary vein stenosis
57 Stent/balloon complication

MISCELLANEOUS

30 Hypertension
40 Diabetic keto acidosis (DKA)
64 Cachexia
66 Malignant disease possibly induced by immunosuppressive therapy—specify primary site ___________________________
67 Malignant disease (not code 66)—specify primary source _________________________
69 Dementia
90 Multi-system failure
99 Other identified cause of death—specify ____

Form 3: Follow-up Hemodialysis form

SEND THIS CONFIDENTIAL INFORMATION TO:
Canadian Organ Replacement Register (CORR)
Canadian Institute for Health Information
4110 Yonge Street, Suite 300
Toronto, Ontario M2P 2B7
Phone: 416-481-2002

Please complete one follow-up form for every living hemodialysis patient being treated at your centre on October 31, 2014.
(Patient label may be attached if same information is provided.)

Hospital Name: ______________________________

Patient Last Name: ______________________________

Patient First and Middle Names: ______________________________

Current Health Card Number: ______________________________

Province of Health Card: ______________________________

Current Postal Code: |___|___|___| |___|___|___|

Date of Birth: |___|___|/|___|___|___|/|___|___|___|___| (DD/MON/YYYY)

Hospital City: ______________________________

Hospital Number: ______________________________

Affix patient label, if available.

1. Provide the details on the latest available laboratory results for this patient. Date cannot exceed December 31, 2014.

**Test**	**Reference Range***	**Laboratory Results**	**Date of Test (MON/YYYY)**	**Test Not Done**
Hemoglobin (g/L) (pre-dialysis)	60–140 g/L	______ g/L	|___|___|___|/|___|___|___|___|	☐
Ferritin (within nearest six months) (pmol/L or µg/L)	50–500 pmol/L<br>Males 14–610 µg/L<br>Females 8–125 µg/L	______<br>☐ pmol/L ☐ µg/L	|___|___|___|/|___|___|___|___|	☐
Iron profile (for example, % saturation, serum iron, transferrin, TIBC)	☐ Iron saturation (25%–50%)<br>☐ Serum iron (9–32 µmol/L) and TIBC (45–81 µmol/L)<br>☐ Serum iron (9–32 µmol/L) and Transferrin (2-0–4.0g/L)	______<br>______<br>______<br>______<br>______	|___|___|___|/|___|___|___|___|	☐
Creatinine (µmol/L) (pre-dialysis)	300–1,500 µmol/L	______ µmol/L	|___|___|___|/|___|___|___|___|	☐
Urea (mmol/L) (pre-dialysis)<br>Urea (mmol/L) (post-dialysis)	15–40 mmol/L<br>5–20 mmol/L	______ mmol/L<br>______ mmol/L	|___|___|___|/|___|___|___|___| *Should be the same date as above.*	☐
☐ Serum bicarbonate (mmol/L) (pre-dialysis) **OR**<br>☐ Serum CO_2 (mmol/L) (pre-dialysis)	20–30 mmol/L	______ mmol/L	|___|___|___|/|___|___|___|___|	☐
Serum calcium (mmol/L) (pre-dialysis)	Various ranges—please specify:<br>☐ 2.10–2.60 mmol/L uncorrected<br>☐ 2.22–2.62 mmol/L corrected<br>☐ 1.19–1.29 mmol/L ionized	______ mmol/L	|___|___|___|/|___|___|___|___|	☐
Serum phosphate (mmol/L) (pre-dialysis)	1.5–1.8 mmol/L	______ mmol/L	|___|___|___|/|___|___|___|___|	☐
Serum parathormone (PTH) (pmol/L; ng/L or pg/ml)	Various ranges—please specify:<br>☐ 1.3–7.6 pmol/L<br>☐ 18–73 ng/L<br>☐ 10–65 pg/ml	______	|___|___|___|/|___|___|___|___|	☐
HbA_{1c} (if patient diabetic)	4%–12% (0.04–0.12)	______ %	|___|___|___|/|___|___|___|___|	☐
Serum albumin (g/L)	25–50 g/L	______ g/L	|___|___|___|/|___|___|___|___|	☐

2. **Is the patient currently receiving erythropoietin? (If patient is temporarily on hold from erythropoietin on October 31 but typically receives it, check "Yes.")**

☐ No ☐ Yes ➔ If yes: Product used: ☐ Eprex ☐ Aranesp ☐ Other:

Route of administration: ☐ IV ☐ Subcutaneously

Frequency of administration: ☐ Weekly ☐ Every two weeks ☐ Every three weeks ☐ Monthly ☐ Other: ______

Total dose within a 7-day period of administration: ______

* *Will depend on laboratory procedures.*

Patient Last Name: ____

Iron Supplementation:

3. a) Is the patient currently on iron?
   ☐ No (1) ☐ Yes ➔ Specify: ☐ Oral (2) ☐ IV (3) ☐ Both (IV and Oral) (4) ☐ Intramuscular (IM) (5) ☐ Other (6)

   b) Has the patient been on iron during the past three months?
   ☐ No (1) ☐ Yes ➔ Specify: ☐ Oral (2) ☐ IV (3) ☐ Both (IV and Oral) (4) ☐ Intramuscular (IM) (5) ☐ Other (6) ☐ On dialysis less than three months (8)

   c) If the patient has been on dialysis for 12 months or more, has the patient been on iron during the past year?
   ☐ No (1) ☐ Yes ➔ Specify: ☐ Oral (2) ☐ IV (3) ☐ Both (IV and Oral) (4) ☐ Intramuscular (IM) (5) ☐ Other (6)

   ☐ On dialysis less than one year (8)

4. a) Patient pre-dialysis weight (kg): |___|___|___|•|___|___|___|

   Patient post-dialysis weight (kg): |___|___|___|•|___|___|___|

   ➔ Date taken: |___|___|/|___|___|___|/|___|___|___|___| (DD/MON/YYYY)

   b) *For pediatric patients only (patients younger than 18):*
   Height (cm): |___|___|___|•|___|___|___|
   ➔ Date taken: |___|___|/|___|___|___|/|___|___|___|___| (DD/MON/YYYY)

   Conversion factors: 1 lb = 0.454 kg; 1 inch = 2.54 cm

5. a) Hemodialysis frequency (treatments per week): _________________

   b) Number of hours per treatment: ______________________________

6. Which access was the patient using on the date the laboratory results were obtained?
   ☐ Temporary catheter non-cuffed (1)
   ☐ Temporary catheter cuffed (2)
   ☐ Permanent catheter non-cuffed (3)
   ☐ Permanent catheter cuffed (4)
   ☐ Fistula (5) ➔ How do you monitor the fistula function in this patient?
   ☐ Not monitored
   ☐ Total access blood flow (1) ➔
   Last flow (mL/min): ______________
   Date: |___|___|___|/|___|___|___|___| (MON/YYYY)
   ☐ Re-circulation (2) ➔
   Last re-circulation (%): ______________
   Date: |___|___|___|/|___|___|___|___| (MON/YYYY)
   ☐ Graft (6) ➔ How do you monitor the graft function in this patient?
   ☐ Not monitored
   ☐ Total access blood flow (1) ➔
   Last flow (mL/min): ______________
   Date: |___|___|___|/|___|___|___|___| (MON/YYYY)
   ☐ Venous pressure (2) ➔
   Last dynamic venous pressure (mmHg) at a blood flow of 200 mL/min: ________
   Date: |___|___|___|/|___|___|___|___| (MON/YYYY)

7. Is the patient currently active on the renal transplant waiting list?
   ☐ No ☐ Yes ☐ Unknown

SEND THIS CONFIDENTIAL INFORMATION TO:
Canadian Organ Replacement Register (CORR)
Canadian Institute for Health Information
4110 Yonge Street, Suite 300
Toronto, Ontario M2P 2B7
Phone: 416-481-2002

Form 4: Follow-up Peritoneal Dialysis form

Please complete one follow-up form for every living hemodialysis patient being treated at your centre on October 31, 2014.
(Patient label may be attached if same information is provided.)

Hospital Name: ____________________

Patient Last Name: ____________________

Patient First and Middle Names: ____________________

Current Health Card Number: ____________________

Province of Health Card: ____________________

Current Postal Code: |___|___|___| |___|___|___|

Date of Birth: |___|___|/|___|___|___|/|___|___|___|___| (DD/MON/YYYY)

Hospital City: ____________________

Hospital Number: ____________________

Affix patient label, if available.

1. Provide the details on the latest available laboratory results for this patient. Date cannot exceed December 31, 2014.

Test	Reference Range*	Laboratory Results	Date of Test (MON/YYYY)	Test Not Done
Hemoglobin (g/L)	60–140 g/L	________ g/L	|___|___|___|/|___|___|___|___|	☐
Ferritin (within nearest six months) (pmol/L or µg/L)	50–500 pmol/L<br>Males 14–610 µg/L<br>Females 8–125 µg/L	________<br>☐ pmol/L ☐ µg/L	|___|___|___|/|___|___|___|___|	☐
Iron profile (for example, % saturation, serum iron, transferrin, TIBC)	☐ Iron saturation (25%–50%)<br>☐ Serum iron (9–32 µmol/L) and TIBC (45–81 µmol/L)<br>☐ Serum iron (9–32 µmol/L) and Transferrin (2-0–4.0g/L)	________<br>________<br>________<br>________<br>________	|___|___|___|/|___|___|___|___|	☐
Creatinine (µmol/L)	300–1,500 µmol/L	________ µmol/L	|___|___|___|/|___|___|___|___|	☐
Urea (mmol/L)	15–40 mmol/L	________ mmol/L	|___|___|___|/|___|___|___|___|	☐
☐ Serum bicarbonate (mmol/L) **OR**<br>☐ Serum CO_2 (mmol/L)	20–30 mmol/L	________ mmol/L	|___|___|___|/|___|___|___|___|	☐
Serum calcium (mmol/L)	Various ranges—please specify:<br>☐ 2.10–2.60 mmol/L uncorrected<br>☐ 2.22–2.62 mmol/L corrected<br>☐ 1.19–1.29 mmol/L ionized	________ mmol/L	|___|___|___|/|___|___|___|___|	☐
Serum phosphate (mmol/L)	1.5–1.8 mmol/L	________ mmol/L	|___|___|___|/|___|___|___|___|	☐
Serum parathormone (PTH) (pmol/L; ng/L or pg/ml)	Various ranges—please specify:<br>☐ 1.3–7.6 pmol/L<br>☐ 18–73 ng/L<br>☐ 10–65 pg/ml	________	|___|___|___|/|___|___|___|___|	☐
HbA_{1c} (if patient diabetic)	4%–12% (0.04–0.12)	________ %	|___|___|___|/|___|___|___|___|	☐
Serum albumin (g/L)	25–50 g/L	________ g/L	|___|___|___|/|___|___|___|___|	☐

2. **Is the patient currently receiving erythropoietin? (If patient is temporarily on hold from erythropoietin on October 31 but typically receives it, check "Yes.")**

☐ No ☐ Yes ➔ If yes: Product used: ☐ Eprex ☐ Aranesp ☐ Other:

Route of administration: ☐ IV ☐ Subcutaneously

Frequency of administration: ☐ Weekly ☐ Every two weeks ☐ Every three weeks ☐ Monthly ☐ Other: ________

Total dose within a 7-day period of administration: ________

* *Will depend on laboratory procedures.*

Patient Last Name: ____

Iron Supplementation:

3. a) Is the patient currently on iron?

☐ No (1) ☐ Yes ➔ Specify: ☐ Oral (2) ☐ IV (3) ☐ Both (IV and Oral) (4)
☐ Intramuscular (IM) (5) ☐ Other (6)

b) Has the patient been on iron during the past three months?

☐ No (1) ☐ Yes ➔ Specify: ☐ Oral (2) ☐ IV (3) ☐ Both (IV and Oral) (4)
☐ Intramuscular (IM) (5) ☐ Other (6)
☐ On dialysis less than three months (8)

c) If the patient has been on dialysis for 12 months or more, has the patient been on iron during the past year?

☐ No (1) ☐ Yes ➔ Specify: ☐ Oral (2) ☐ IV (3) ☐ Both (IV and Oral) (4)
☐ Intramuscular (IM) (5) ☐ Other (6)
☐ On dialysis less than one year (8)

4. a) Patient weight (kg) at a clinic attendance: |___|___|___|•|___|___|___|

➔ Patient is: ☐ Empty of PD fluid (0) ☐ Full of PD fluid (1)

➔ Date taken: |___|___|/|___|___|___|/|___|___|___|___| (DD/MON/YYYY)

b) *For pediatric patients only (patients younger than 18):*
Height (cm): |___|___|___|•|___|___|___|

➔ Date taken: |___|___|/|___|___|___|/|___|___|___|___| (DD/MON/YYYY)

Conversion factors: 1 lb = 0.454 kg; 1 inch = 2.54 cm

5. a) Weekly creatinine clearance (L/1.73 m^2/week)

Residual renal (R) ________________
Peritoneal (P) ________________
Total (R + P) ________________
Date taken: |___|___|/|___|___|___|/|___|___|___|___| (DD/MON/YYYY)

☐ Patient not yet tested ☐ Not routinely done

b) Weekly Kt/V (Urea)

Residual renal (R) ________________
Peritoneal (P) ________________
Total (R + P) ________________

Date taken: |___|___|/|___|___|___|/|___|___|___|___| (DD/MON/YYYY)

☐ Patient not yet tested ☐ Not routinely done

c) Peritoneal membrane transport status
(Please use results of first PET.)

☐ Low (1) ☐ Low Average (2)
☐ High (3) ☐ High Average (4)

☐ Patient not yet tested ☐ Patient declined test
☐ Test not routinely done

6. Type of peritoneal dialysis:

☐ CAPD

(Includes manual exchanges. It can also include the use of a night exchange device to do one automated exchange per 24 hours.
If more than one automated exchange is done, it should be considered APD.)

If CAPD ➔ Volume of fluid per exchange (mL): ______
➔ Number of exchanges per day: ________
➔ Total volume per day (mL): ________
➔ Is a night exchange device used?

☐ No ☐ Yes

☐ APD *(includes all other types of PD)*

If APD ➔ Volume cycled per night (mL):______
➔ Dwell volume on cycler (mL):______
➔ Volume of individual day dwells (mL):_____
➔ Number of day dwells:______

7. Is this patient using amino acid dialysate?

☐ No ☐ Yes

8. Is this patient using non-dextrose (that is, icodextrin, no amino acid added) dialysate?

☐ No ☐ Yes

9. Is the patient currently active on the renal transplant waiting list?

☐ No ☐ Yes ☐ Unknown

SEND THIS CONFIDENTIAL INFORMATION TO:
Canadian Organ Replacement Register (CORR)
Canadian Institute for Health Information
4110 Yonge Street, Suite 300
Toronto, ON M2P 2B7
Tel: 416-481-2002

Form 5: Facility Profile Hemodialysis form

Complete this form to reflect the situation at your facility on December 31, 2010. Please keep a copy for your records.

Hospital Name: ______________________ Hospital City: ____________________________

SECTION A—FACILITY RESOURCES Hospital Number : _______________

1. Does your facility re-use dialysers?

☐ No ☐ Yes ➔ What type of system is used?
☐ Manual (M) ☐ Automated (A)

➔ What number patients were on re-used dialysers on December 31, 2010? ________

➔ What method is used for sterilizing re-used dialysers? *(Please check one.)*

☐ Heat (H)
☐ Formaldehyde (F)
☐ Renalin (R)
☐ Glutaraldehyde (G)
☐ Other chemical (specify) (O)
____

2. a) What is the total number of haemodialysis stations at your facility?
____
(Note: This does not include stations at community centres.)

b) On page 3 of this questionnaire, please list all the community centres affiliated with your facility and the number of stations at each centre.

3. Is continuous renal replacement therapy (CRRT) used?

☐ No (0) ☐ Yes ➔ Who is responsible for CRRT?
☐ Nephrologist only (1) ☐ Intensivist only (2)
☐ Nephrologist/Intensivist share care (3)

4. a) How many haemodialysis patients are being dialysed using the following accesses? *(Please report the number of patients.)*

	At Hospital	At Home	At Community Centre
Natural vein fistula	________	________	________
Synthetic arteriovenous graft (PTFE)	________	________	________
Saphenous vein graft	________	________	________
Permanent central venous catheters	________	________	________
Temporary subclavian vein catheters	________	________	________
Temporary internal jugular vein catheters	________	________	________
Temporary femoral vein catheters	________	________	________
Other (specify) _________________			
4. b) How many haemodialysis patients are on more than one access on December 31, 2010?	________	________	________

5. a) Does your facility provide dialysis facilities to temporary visitors (including holidays)?

☐ No ☐ Yes

b) Is your facility always able to provide dialysis facilities to temporary visitors?

☐ No ☐ Yes

6. a) Does your facility have adequate haemodialysis facilities (e.g., in terms of space/physical capacity, human resources)?

☐ No ➔ If not, select the **top 2** reasons why facilities are inadequate: ☐ Yes

☐ inadequate space for patients (1)
☐ inadequate space for machines (2)
☐ inadequate space for training facilities (3)
☐ lack of physical capacity to expand (4)
☐ lack of qualified registered nurses(5)
☐ lack of dieticians, social workers, pharmacists or other allied health professionals (6)
☐ lack of technicians/technologists (7)
☐ lack of dedicated nephrologist(s) (8)
☐ other—please specify : ____________(9)

b) Do staff and patients have free choices as to which modality is selected?

☐ No ➔ If not, select the **top 2** reasons why patients do not have free choice: ☐ Yes

☐ other modality not supported at centre (1)
☐ space restrictions limit options (2)
☐ geographic access to centre by patients limits options (3)
☐ other—please specify : ______________(9)

Canadian Institute for Health Information
Institut canadien d'information sur la santé

Formulaire disponible en français.

Form FP-H2010

Hospital Name: ___________________________

SECTION B—TREATMENT AVAILABLE

7. a) What types of haemodialysis treatment are supported by your facility? *(Please check all that apply.)*

	Acute Care Hospital			Community Centre			Chronic Care Hospital	Home
	Total Care	Limited Self Care	Total Self Care	Total Care	Limited Self Care	Total Self Care	Total Care	Total Self Care
Conventional HD	☐	☐	☐	☐	☐	☐	☐	☐
Short Daily HD	☐	☐	☐	☐	☐	☐	☐	☐
Slow Nocturnal HD	☐	☐	☐	☐	☐	☐	☐	☐

b) Please indicate the number of patients on each form of treatment as of December 31, 2010

	Acute Care Hospital			Community Centre			Chronic Care Hospital	Home
	Total Care	Limited Self Care	Total Self Care	Total Care	Limited Self Care	Total Self Care	Total Care	Total Self Care
Conventional HD	______	______	______	______	______	______	______	______
Short Daily HD	______	______	______	______	______	______	______	______
Slow Nocturnal HD	______	______	______	______	______	______	______	______

SECTION C—ADEQUACY/CLEARANCE

8. a) Is urea kinetic modelling used to monitor haemodialysis prescription?

☐ No ☐ Yes ➔ What percentage of patients are routinely monitored?

☐ 0–49% of patients (0)
☐ 50–75% of patients (1)
☐ 76–100% of patients (2)

➔ What is the target Kt/V or percent reduction in the urea (PRU)?

Kt/V	PRU
☐ 1.2 to 1.4 (0)	☐ 0 to 64% (0)
☐ Over 1.4 (1)	☐ 65 to 69% (1)
☐ Other (specify)____ (2)	☐ 70 to 74% (2)
	☐ 75% and over (3)

➔ How frequently is urea kinetic modelling used per haemodialysis patient? *(Please approximate by location.)*

	(0) Once per mo.	(1) Once every 3 mos.	(2) Once every 6 mos.	(3) Once every 12 mos.	(4) Other
Acute Care Hospital	☐	☐	☐	☐	☐
Community Centre	☐	☐	☐	☐	☐
Chronic Care Hospital	☐	☐	☐	☐	☐
Home	☐	☐	☐	☐	☐

SECTION D—NURSING HOME/CHRONIC CARE

9. How many haemodialysis patients reside or are awaiting placement in a nursing home or chronic care facility?

patients residing in a nursing home _________
patients awaiting placement in a nursing home _________
patients residing in a chronic care facility _________
patients awaiting placement in a chronic care facility _________

Nursing home: A facility where residents require personal care assistance and/or assistance with activities of daily living.
Chronic Care Facility: A facility where, due to the health needs of residents, ongoing medical intervention is provided.

10. Does your facility have a home haemodialysis training program?

☐ No ☐ Yes ➔ How many home patients were trained for home haemo during the year 2010? ________________

➔ Do you have dedicated home training station(s)?

☐ No ☐ Yes ➔ How many? ______

11. How many home haemodialysis patients are assisted with their dialysis by a paid assistant? ________________

12. How many haemodialysis patients were receiving erythropoietin on December 31, 2010?
_____ Eprex _____ Aranesp _____ Other—please specify:

13. How many haemodialysis patients were receiving growth hormone on December 31, 2010? *(For paediatric patients only.)*
____

SECTION E—SCREENING

14. Does your facility screen new haemodialysis patients for:

Hepatitis B? ☐ No ☐ Yes
Hepatitis C? ☐ No ☐ Yes

15. Are haemodialysis patients routinely vaccinated against Hepatitis B?

☐ No ☐ Yes

16. Does your facility have an isolation room for patients who require isolation?

☐ No ☐ Yes

17. a) Which of the following best describes the policy of your facility regarding HIV antibodies (HTLV-III/LAV) among haemodialysis patients with end-stage renal disease? *(Please check all that apply.)*

☐ All patients are tested
☐ New patients are tested
☐ Only patients with specific indications are tested
☐ No patients are tested

b) How many haemodialysis patients in your program were HIV positive in 2010? _______

c) How many haemodialysis patients in your program died due to AIDS in 2010? _______

Formulaire disponible en français.

Hospital Name: ____________________________

List of Community Centres *(See question 2b on page 1.)*

	Community Centre Name/Satellite Unit Name	Location	# of Stations
(1)			
(2)			
(3)			
(4)			
(5)			
(6)			
(7)			
(8)			
(9)			
(10)			
(11)			
(12)			
(13)			
(14)			
(15)			
(16)			
(17)			
(18)			
(19)			
(20)			

Completed by: ____________________________ Date: ____________________________

Print Name: ____________________________ Tel.: ____________________________

Fax: ____________________________ Email: ____________________________

Name of contact person *(if different from above)*: ____________________________

Tel.: ____________________________ Fax: ____________________________

Email: ____________________________

In which language would you prefer to receive feedback? ☐ English ☐ French

Thank you for completing this questionnaire. Please take a few moments to ensure that all the questions are answered.

Form 6: Facility Profile Peritoneal Dialysis form

SEND THIS CONFIDENTIAL INFORMATION TO:
Canadian Organ Replacement Register (CORR)
Canadian Institute for Health Information
4110 Yonge Street, Suite 300
Toronto, ON M2P 2B7
Tel: 416-481-2002

Complete this form to reflect the situation at your facility on December 31, 2010. Please keep a copy for your records.

Hospital Name: ______________________

Hospital City: ____________________________

Hospital Number: ______________

SECTION A—FACILITY RESOURCES

1. a) Does your facility have adequate peritoneal dialysis facilities (e.g., in terms of space/physical capacity, human resources)?

☐ No ➔ If not, select the **top 2** reasons why facilities are inadequate. ☐ Yes

☐ inadequate space for patients (1)
☐ inadequate space for machines (2)
☐ inadequate space for training facilities (3)
☐ lack of physical capacity to expand (4)
☐ lack of qualified registered nurses (5)
☐ lack of dieticians, social workers, pharmacists or other allied health professionals (6)
☐ lack of technicians/technologist(s) (7)
☐ lack of dedicated nephrologist(s) (8)
☐ other—please specify: ______________________________ (9)

b) Do staff and patients have free choices as to which modality is selected?

☐ No ➔ If, not select the **2 top** reasons why patients do not have free choices ☐ Yes

☐ other modality not supported at centre (1)
☐ space restrictions limit options (2)
☐ geographic access to centre by patients limits options (3)
☐ other—please specify: ______________________________ (9)

SECTION B—TREATMENT AVAILABLE

2. a) What types of peritoneal dialysis treatment are supported by your facility? *(Please check all that apply.)*

PD combined with haemodialysis ☐

	Acute Care Hospital	Chronic care Hospital	Home
CAPD (Continuous Ambulatory)	☐	☐	☐
APD (Automated PD)	☐	☐	☐

b) Please indicate the number of patients on each form of treatment as of December 31, 2010.

PD combined with haemodialysis ______

	Acute Care Hospital		Chronic care Hospital		Home
	Total Care	Limited Care	Total Care	Limited Care	
CAPD (Continuous Ambulatory)	_____	_____	_____	_____	_____
APD (Automated PD)	_____	_____	_____	_____	_____

SECTION C—ADEQUACY/CLEARANCE

3. a) Are Peritoneal Equilibration Tests (PETs) done on PD patients?

☐ No ☐ Yes ➔ What proportion of patients have a PET within three months of initiating PD?

☐ < 25% of patients (0)
☐ 25–50% of patients (1)
☐ 51–75% of patients (2)
☐ 76–100% of patients (3)

b) Are adequacy/clearance measurements done on PD patients?

☐ No ☐ Yes ➔ What proportion of PD patients have Kt/V urea or creatinine clearance done at least once a year?

☐ < 25% of patients (0)
☐ 25–50% of patients (1)
☐ 51–75% of patients (2)
☐ 76–100% of patients (3)

➔ Which of the following are routinely done and how many times during the year are they usually done?

24-hr dialysate creatinine clearance
☐ No ☐ Yes ➔ # times per year: ____

24-hr urinary creatinine clearance
☐ No ☐ Yes ➔ # times per year: ____

24-hr dialysate Kt/V urea
☐ No ☐ Yes ➔ # times per year: ____

24-hr urinary Kt/V urea
☐ No ☐ Yes ➔ # times per year: ____

c) Is this information used within a computer modeling program to validate the initial (i.e., within three months) peritoneal dialysis prescription?

☐ No ☐ Yes ➔ What program? (specify) _____________
____

SECTION D—NURSING HOME/CHRONIC CARE

4. How many peritoneal dialysis patients reside or are awaiting placement in a nursing home or chronic care facility?

   # patients residing in a nursing home ________
   # patients awaiting placement in a nursing home ________
   # patients residing in a chronic care facility ________
   # patients awaiting placement in a chronic care facility ________

Nursing home: A facility where residents require personal care assistance and/or assistance with activities of daily living.
Chronic Care Facility: A facility where, due to the health needs of residents, ongoing medical intervention is provided.

5. Does your facility have a home PD training program?

   ☐ No ☐ Yes ➔ How many home patients were trained for home peritoneal dialysis during the year 2010? ________

6. How many PD patients were receiving erythropoietin on December 31, 2010?

   _____ Eprex _____ Aranesp _____ Other—please specify:

7. How many PD patients were receiving growth hormone on December 31, 2010? *(For paediatric patients only.)* ____________

SECTION E—SCREENING

8. Does your facility screen new PD patients for:

   Hepatitis B? ☐ No ☐ Yes
   Hepatitis C? ☐ No ☐ Yes

9. Are PD patients routinely vaccinated against Hepatitis B?

   ☐ No ☐ Yes

10. Does your facility have an isolation room for patients who require isolation?

    ☐ No ☐ Yes

11. a) Which of the following best describes the policy of your facility regarding testing of HIV antibodies (HTLV-III/LAV) among PD patients with end-stage renal disease?
    (Please check all that apply.)

    ☐ All patients are tested
    ☐ New patients are tested
    ☐ Only patients with specific indications are tested
    ☐ No patients are tested

    b) How many PD patients on dialysis in your program were HIV-positive in 2010? _______

    c) How many PD patients in your program died due to AIDS in 2010? _______

Completed by: ____________________

Date: ____________________

Print Name: ____________________

Telephone: ____________________

Fax: ____________________

Email: ____________________

Name of contact person *(if different from above):*

____

Telephone: ____________________

Fax: ____________________

Email: ____________________

In which language would you prefer to receive feedback?

☐ English ☐ French

Thank you for completing this questionnaire. Please take a few moments to ensure that all the questions are answered.
